# Using responsive evaluation to shape research: Engaging and collaborating with stakeholders in the international symposium on multimorbidity

**DOI:** 10.1177/26335565251388513

**Published:** 2025-10-31

**Authors:** Nina Grede, Christiane Muth, Maria Hanf, Amaia Calderón-Larrañaga, José Maria Valderas, Svetlana Puzhko, Marjan van den Akker

**Affiliations:** 1 Institute of General Practice, Goethe University, Frankfurt am Main, Germany; 2 General Practice and Family Medicine, Medical School East Westphalia, 9167 Bielefeld University, Bielefeld, Germany; 3 Department of Neurobiology, Care Sciences and Society, Karolinska Institutet and Stockholm University, 193201 Aging Research Center, Stockholm, Sweden; 4 Stockholm Gerontology Research Center, Stockholm, Sweden; 5 Department of Family Medicine, 150744 National University Health System, Singapore

**Keywords:** multimorbidity, interdisciplinary collaboration, stakeholder engagement, individualized care, health policy, scientific conference

## Abstract

**Background:**

Multimorbidity (MM) is a growing global public health issue requiring interdisciplinary collaboration among researchers, healthcare professionals, policymakers, and patients. The third International Symposium on Multimorbidity, held in May 2024 in Bielefeld, Germany, provided a platform for knowledge exchange and stakeholder engagement to address key challenges in MM research and care.

**Methods:**

The symposium followed a structured responsive evaluation approach with continuous stakeholder involvement. A pre-symposium survey was distributed to 116 international experts in the potential target audience; 85 responded, identifying the most pressing topics in MM. The lack of direct patient and policy maker involvement may have influenced the prioritization of certain topics.

**Results:**

Key topics included medication management, coordinated and personalized care, methods for measuring MM, applications of artificial intelligence, and concerns regarding overdiagnosis and overtreatment. These results informed the symposium agenda, ensuring relevance to the professional community. Participants from 16 countries attended, reflecting widespread international interest. A post-symposium survey (response rate: 19%) indicated high satisfaction; 85.5% of respondents would recommend the event. Feedback highlighted the need for broader topic coverage, more practical applications, and enhanced networking opportunities. Limitations included the low response rate for the post-symposium survey and potential self-selection bias.

**Conclusion:**

The symposium effectively facilitated discussion and knowledge exchange through a structured, stakeholder-driven format. Recommendations for future events include expanding topic variety, integrating practical components, improving logistics, and incorporating real-time feedback tools. These insights support ongoing advancements in MM research, policy, and clinical practice, emphasizing the importance of interdisciplinary and participant-centered approaches in academic event planning.

## Introduction

Multimorbidity (MM), commonly defined as the coexistence of two or more chronic conditions, has become the norm rather than the exception in primary care, with profound implications for healthcare delivery, research, and policy.^1^ It is increasingly recognized as a central issue in public health, requiring interdisciplinary collaboration and new models of care.^2^

As the prevalence of MM continues to rise globally, it has become increasingly crucial to engage a diverse array of stakeholders—including researchers, healthcare professionals, policymakers, and patients—in multidisciplinary conversations that address the multifaceted challenges presented by this significant public health concern. It is increasingly recognized as a central issue in public health, requiring interdisciplinary collaboration and new models of care^3^

In May 2024, the third edition of the International Symposium on Multimorbidity took place in Bielefeld (Germany), marking a relevant milestone in the ongoing discourse surrounding the complexities of the provision of care for patients with multiple chronic conditions. The symposium aimed to foster an environment of collaboration and knowledge exchange among leading experts and stakeholders from various disciplines. By creating a forum for dialogue, the event sought, not only to disseminate up-to-date scientific knowledge, but also to stimulate critical discussions on best practices, potential interventions, and emerging trends in the field of MM.

Recognizing that the effective management of MM requires a multidisciplinary approach, the symposium’s agenda was designed to encompass a wide range of topics, reflecting the diverse perspectives and expertise of its participants. Addressing these challenges requires not only robust research but also careful prioritization of topics that reflect both clinical realities and stakeholder needs.^4^ Considering these objectives, the organizing committee undertook a comprehensive planning process to ensure that the symposium would effectively meet the needs and interests of its audience. This involved identifying relevant themes, engaging experts, and utilizing a responsive evaluation framework to guide the planning and execution of the event. By prioritizing stakeholders’ engagement and incorporating their feedback throughout the planning stages, the committee focused on developing a program that was both scientifically robust and highly relevant to the participants’ clinical and scientific experiences and concerns.

The symposium sought not only to enhance knowledge and understanding of the topic but also to build a community of practice that could drive forward improved research, policy, and clinical practice in this field. As such, this symposium served as a vital opportunity for networking, collaboration, and sharing insights that could lead to improved outcomes for individuals living with multiple chronic conditions.

Building on the insights and results of the symposium, the objectives of this paper are to provide a comprehensive overview of the scientific sessions, key discussions, stakeholder engagement strategies, and thematic priorities, and to describe the iterative process of planning the symposium, including how stakeholder feedback influenced topic selection. Through reflection on the responsive evaluation approach employed, the paper also aims to identify lessons learned and to offer recommendations for advancing MM research, policy, and practice. This paper combines a description of the planning process with a reflection on stakeholder feedback and outcomes, positioning it between a protocol and an evaluative report.

## Methods

The interdisciplinary organizing committee (Christiane Muth, Svetlana Puzhko, Marjan van den Akker, Jose M Valderas, Amaia Calderón-Larrañaga, Paul Glasziou and Maria Hanf) aspired to design a compelling and scientifically sound program. Particular emphasis was placed on selecting topics that reflect current developments in MM and can capture the interest of a wide and multidisciplinary professional audience. Table 1 summarizes key organizational elements and planning steps to illustrate the structure and scope of the responsive evaluation process used.

**Table 1.** Key organizational elements of the symposium.

Description	Details
Title	Ariadne revisited: Gains and gaps in research and care – 3rd international symposium on multimorbidity
Date	May 3, 2024
Location	Bielefeld University, Germany
Total attendees	Approximately 100 participants from 16 countries
Format of sessions	Keynote lectures, plenary discussions, abstract presentations, and interactive panels
Accessibility	In-person only
Key topics	Artificial intelligence in MM, interventions, and health system redesign
Organized by	Bielefeld University (faculty of medicine), Goethe University Frankfurt (institute of general practice)
Target audience	Researchers, clinicians, healthcare professionals, policymakers

More information: https://www.uni-bielefeld.de/fakultaeten/medizin/fakultaet/arbeitsgruppen/allgemeinmedizin/multimorbidity-symposium/programme/ubersicht/.

In the planning and execution of the symposium, we adopted the principles of responsive evaluation. This adaptive and participatory approach, which emphasizes ongoing engagement with stakeholders throughout the evaluation process, was originally proposed by Stake^5^ for evaluating educational programs and later extended by Guba and Lincoln through enhancement of the underlying theoretical framework.^6^ Abma further developed this methodology for the public health field, incorporating more interactive and participatory elements to better address community needs.^7^

In contrast to traditional evaluation approaches that focus on predefined outcomes or success indicators, responsive evaluation prioritizes stakeholder perspectives, contextual understanding, and iterative learning. It is particularly suitable for complex settings where goals may evolve over time and multiple viewpoints need to be considered.

Involvement of stakeholders in the early planning stages was directed at identifying and prioritizing the most relevant topics for the symposium. The iterative process of collecting inputs, analyzing responses, and refining the program was integral to crafting a symposium that not only addressed the latest scientific developments but also resonated with the diverse interests of our participants. The dynamic exchange between the organizing committee and stakeholders was crucial for maintaining relevance and engagement throughout the event.^6,8^ This approach further underscores the importance of stakeholder-centric planning in achieving the symposium’s objectives.

As the first step in selecting symposium topics, we listed and categorized topics from recent publications in the field of MM, using the publication repository (updated on February 8, 2023) of the School of Health and Wellbeing, University of Glasgow (https://www.gla.ac.uk/schools/healthwellbeing/research/generalpractice/internationalmultimorbidity/publications). This repository was selected because of its comprehensive focus on multimorbidity and its continuous and structured updates, which provided a broad overview of current research. We further complemented our review with a targeted PubMed search focusing on recent and underrepresented areas in the multimorbidity literature. Initially, a brief PubMed search was conducted without time restrictions in order to identify relevant reviews and emerging themes. This search was later refined to include only studies published between 2019 and 2022, focusing on themes such as “choosing wisely,” “deprescribing” (which could be categorized under “medication”), “overlap multimorbidity/frailty/disability,” “overdiagnosis,” “AI,” and “core outcome sets.” By melding both approaches, we wanted to address their respective limitations while ensuring feasibility within the available resources and capturing emerging and relevant themes in MM. This process helped to identify a broad range of potential topics and categories and then provide an overview of the current landscape in the field. The initial results were further refined through several rounds of structured discussions within the organizing committee. A consensus-based approach was used, whereby potential topics were reviewed and prioritized according to their perceived relevance, novelty, and feasibility. Committee members provided individual feedback, followed by group deliberation to reach agreement. This iterative process ensured that final topic selections reflected both stakeholder input and expert judgment. This approach helped to narrow down the list of topics and prioritize those most relevant to the symposium’s goals, particularly concerning research and clinical practice.

A questionnaire including a wide list of topics linked to MM was subsequently developed, which underwent several revisions by the committee through an iterative process of internal review and discussion, ensuring clarity, feasibility, and alignment with the preferred topics of the target audience. The resultant questionnaire was implemented online through LimeSurvey in both German and English to gather insights from potential symposium participants. This survey was designed to identify the pre-defined topics that would motivate attendees to travel to the symposium and to obtain more specific information on their professional backgrounds and participation preferences. The questionnaire consisted of these five items.

1. Participants were asked to mark the five most important items out of 22 listed themes that would motivate them to visit the symposium. The topics varied from “biological and genetic markers” to “medical education”, and “multimorbidity in low and middle income countries” to methodological issues;
2. An open question invited participants to suggest further topics of interest;
3. Participant availability to visit the symposium at different times was assessed;
4. The professional background of the participants (scientist, GP/family physician, other physician, nurse, other healthcare professional, policy maker, health manager, student, or other background) was recorded;
5. Information on working discipline of the participants (family medicine, internal medicine, geriatrics, health sciences, public health, nursing, pharmacy and/or other areas) was collected.

The questionnaire was distributed to an international group of experts, researchers, and practitioners in the field of MM who were invited to participate through issuance of individualized invitations, sent personally by the members of the organizing committee via email lists compiled from previous conferences, professional societies, and personal networks. Additionally, we encouraged the invitees to forward our email within their own networks to maximize our survey reach and to engage a more diverse group of participants, spanning those in a larger variety of professions, such as scientists, physicians, healthcare professionals, policy makers, health managers, and also students.

A short seven-question survey was additionally administered to the participants at the end of the symposium, covering the following themes: likelihood of recommending the symposium to others, relevance of symposium topics to professional interests, coverage of topics, clarity and coherence of the presentations, timeline and structure of the symposium. Five questions used a three-point Likert scale, while the general impression was measured using a visual analogue scale, and further comments were asked for as an open-ended question. The paper-based survey was conducted anonymously and distributed among participants and collected immediately following the event. No personal data were collected. No reminders or incentives were used.

Responses were analyzed using descriptive statistics for closed-ended questions and thematic coding for open-ended responses. Frequencies were calculated for topic priorities, and qualitative comments were grouped into thematic categories by the organizing team.

## Results

### Pre-symposium survey

In June 2023, a total of 116 individuals received the link to the online questionnaire. Of those, N=85 filled out the questionnaire (N=67 English questionnaire; N=18 German questionnaire). The diverse range of respondents included scientists (70%), general practitioners (25%), other physicians (15%), healthcare professionals (21%) and nurses (3%), with multiple responses allowed, emphasizing the multidisciplinary nature of the target group. Respondents represented 16 countries, including Australia, Belgium, Canada, China, Denmark, France, Germany, Ireland, Italy, the Netherlands, Singapore, Spain, Sweden, Switzerland, the United Kingdom, and the United States, further highlighting the international and multidisciplinary nature of the target group.

The survey revealed several key topics of interest among participants, reflecting the pressing issues in the context of MM (Table 2). The table presents the absolute number of participants who selected each topic, as participants were able to choose multiple topics. We decided to use the absolute numbers rather than percentages to provide a clearer view of the responses and avoid any potential confusion from multiple selections.

**Table 2.** Key topics selected by pre-symposium survey respondents.

Topic	Response n (%)
Medication (e.g., polypharmacy, deprescribing, choosing wisely, medication adherence, interactions)	39 (45.9)
Coordinated, personalized, integrated care (incl. continuity of care, goal-oriented care, care transitions, interprofessional care)	36 (42.4)
Methods to measure MM and co-morbidity, definition, conceptualization, overlap MM, frailty, disability	32 (37.6)
Application of artificial intelligence	31 (36.5)
Overdiagnosis, overtreatment, treatment burden	31 (36.5)
Patient preferences and shared decision-making	8 (9.4)
Digital medicine, e-health, telemedicine	6 (7)
Generalization of clinical trials, guidelines	5 (5.9)

The key topics presented reflect the thematic priorities identified in the pre-symposium survey, rather than the exact session titles. This approach allowed for broader themes to be addressed across multiple sessions and formats during the symposium.

Medication management was the most prominent topic of interest, including aspects such as polypharmacy, deprescribing strategies, and medication adherence. Following closely, the importance of coordinated, personalized, and integrated care was highlighted. Another significant area of interest was that of methods for measuring MM and co-morbidity, reflecting a growing concern for establishing clear definitions and conceptual frameworks within the field. Emerging technologies, particularly the application of artificial intelligence, also garnered considerable attention, showcasing a strong interest in innovative solutions to enhance healthcare delivery for individuals. The interest in the topics of overdiagnosis, overtreatment, and treatment burden further illustrate the complexities and challenges faced by healthcare providers in managing multiple chronic conditions.

Topics such as patient preferences, shared decision-making, cost of health care, and digital medicine received fewer responses, most likely due to the lack of direct patient and policy maker involvement in the pre-symposium survey. While these topics were therefore less prioritized in the agenda, they remain essential areas for future exploration—particularly in enhancing patient engagement and the use of technology in care delivery—highlighting the need to integrate patient and policy maker perspectives more systematically in future symposia planning processes.

The final program can be found at: https://www.uni-bielefeld.de/fakultaeten/medizin/fakultaet/arbeitsgruppen/allgemeinmedizin/multimorbidity-symposium/programme/ubersicht/.

### Post-symposium survey

The post-symposium survey was filled out by 19 out of 100 participants of the symposium. The majority of respondents identified as scientists (77.8%) and general practitioners (72.2%), with overlapping roles due to the multiple-choice format. Other stakeholder categories, such as policymakers, healthcare managers, students, or nurses, were not represented among respondents. Results indicate positive reception of the symposium, with high scores on key aspects such as the likelihood of recommending the event and the alignment of topics with participants’ professional interests. However, aspects linked to topic coverage and the request for additional practical insights were scored lower, strongly pointing to areas for potential improvement in future events. Feedback specifically emphasized the need for improved networking opportunities. Participants also noted challenges related to session overlaps, limited hybrid accessibility, and unclear event communication. These logistic aspects were highlighted as areas for improvement in open-ended responses. Additional comments also indicated interest in more hands-on formats and broader thematic scope for future symposia.

Due to the concentration of responses within few professional roles and/or stakeholder groups, subgroup comparisons were not feasible and are acknowledged as a limitation.

## Discussion

The surveys conducted before and after the International Symposium on MM provided valuable insights into the effectiveness of the symposium’s planning and execution. They also highlight the key areas of interest for participants and potential avenues for future improvements.

The pre-symposium survey significantly influenced the agenda and content of the symposium. The high level of participation in the pre-survey and the focus on practical, actionable topics suggest that the attendees are not only seeking to expand their knowledge but are also looking for solutions to the challenges they face in their practice. By soliciting input from potential attendees on the most pressing topics in the context of MM, the organizers ensured that the symposium addressed the needs and interests of its target audience. The top five topics identified—medication management, coordinated and personalized care, methods for measuring MM, application of artificial intelligence, and concerns about overdiagnosis and overtreatment—clearly reflect the current challenges and priorities in the field.

The pre-symposium survey was distributed amongst an international group of stakeholders, with responses reflecting diverse geographic representation. The symposium ultimately attracted participants from 16 countries. Despite Bielefeld`s relatively limited accessibility, its relatively young medical faculty, and its less prominent status as a tourist destination, a significant number of national and international participants nevertheless travelled to attend the symposium. This underscores the strong interest in the topics covered and the event’s perceived value within the professional community. This global reach highlights the wide-ranging appeal of the topics selected through the survey and the relevance of the event to a broad professional audience. Comparable participation data from previous symposiums were not systematically documented, which limits our ability to evaluate changes in international reach or stakeholder diversity over time. Future events may benefit from consistent tracking of such metrics. The post-symposium survey results further validate the effectiveness of the content selection process. With an average recommendation likelihood score of 85.5%, it is clear that the symposium was well-received.

These findings suggest that responsive evaluation can serve as a valuable model for organizing interdisciplinary academic events, particularly in complex fields like multimorbidity. It promotes relevance, inclusion, and actionable outcomes, which are essential for advancing both research and clinical practice.

While the findings are rooted in a specific symposium context, many of the insights—such as the value of responsive evaluation and early stakeholder involvement—are transferable to other academic conferences, particularly those addressing complex, interdisciplinary health challenges. This approach can enhance relevance, engagement, and practical impact beyond the field of multimorbidity.

## Limitations

While the use of pre- and post-symposium surveys allowed for a comprehensive understanding of participant interests and feedback, there are limitations to this approach that need to be considered.

• One of the key challenges encountered was the low response rate of the post-symposium survey. Despite a relatively high initial turnout at the symposium, only 19 out of 100 participants completed the survey. This limits the representativeness and reliability of the feedback and may introduce response bias, as respondents might have had particularly strong opinions. To improve future response rates and feedback quality, organizers should consider offering multiple formats (e.g., digital and paper versions), sending reminders, and providing small incentives for participation.
• Both the pre- and post-surveys suffered from some level of **self-**selection bias. The people who responded may have had particularly strong opinions or interests in the symposium’s content, and their feedback may not be fully representative of the broader attendee population. In the future, strategies could be developed to mitigate this bias, such as repeated reminders or providing incentives for post-symposium feedback.
• While responsive evaluation serves as a critical tool for ensuring that assessments are both useful and relevant to stakeholder needs, it is important to acknowledge the limitation that not all segments of the broader community, such as patients, were included in this process. Topics such as patient preferences, shared decision-making, and digital medicine received fewer responses, most likely due to the lack of direct patient involvement in the pre-symposium survey. Still, these topics represent essential areas for future exploration, particularly in enhancing patient engagement and the use of technology in care delivery, and further reinforce the need to integrate patient perspectives more systematically in symposia planning processes.
• The lack of diversity in stakeholder subgroups limits the ability to assess whether topic preferences differed between professions or disciplines.

## Learning points and recommendations

### Responsive evaluation as a continuous process

The success of the symposium highlights the importance of integrating responsive evaluation not as a single intervention but as an iterative and continuous process. This approach ensures that both immediate and long-term participant needs are addressed, fostering a sense of inclusivity and relevance.

### Broadening the scope of topics

Participant feedback revealed a strong demand for a more diverse range of topics, including emerging trends and interdisciplinary connections. Specific suggestions mentioned in the open-ended survey responses included the integration of mental health in multimorbidity care, the use of digital health tools for patient monitoring, and the intersection of multimorbidity with social determinants of health. Future symposia could benefit from expanding their thematic focus to cover such novel areas that align with the evolving interests of the academic and professional communities, when time and finances allow. However, broadening the scope and integrating additional practical workshops would require significant additional resources, including extended program time, increased venue and staffing costs, and higher travel expenses for speakers.

### Practical application of knowledge

One recurring theme in the feedback was the desire for more actionable insights and practical sessions. Future symposia might consider integrating hands-on workshops, case studies, and/or panel discussions that bridge theory and practice, allowing participants to directly apply what they have learned. For example, practical sessions could include interactive formats such as small-group simulations of clinical decision-making for multimorbid patients, moderated case discussions involving both clinicians and researchers, or collaborative exercises on implementing deprescribing tools. These formats would help translate research findings into everyday practice and foster interdisciplinary exchange.

### Improving accessibility and logistic aspects

While the symposium received positive reviews, participants also pointed to areas where logistic improvements could enhance the overall experience. Recommendations include optimizing the schedule to avoid overlaps between key sessions, offering hybrid attendance options for greater accessibility, and ensuring clear communication of event details.

### Enhancing networking opportunities

Feedback from attendees emphasized the value of informal interactions and networking opportunities. Building more structured networking events, such as roundtables or thematic breakout sessions, could foster meaningful connections among participants.

### Real-time feedback

Moving forward, it will be essential to maintain flexibility and adaptability in planning future symposia. The organizers should consider creating mechanisms for real-time feedback during the event, enabling them to make on-the-fly adjustments as needed. Additionally, leveraging technology, such as AI-driven data analysis tools, could help identify subtle trends in participant feedback and inform decisions for subsequent events.

### Lessons from symposium planning

Organizing the symposium highlighted the importance of early stakeholder engagement, clear communication within the planning team, and flexibility in responding to participant needs. Balancing scientific rigor with logistic feasibility was essential, particularly in an interdisciplinary and international context.

### Continuity and accessibility of future symposia

To maintain momentum in the field of multimorbidity, regular organization of symposia—ideally on a biennial basis—should be considered. Hosting future events in a variety of international locations, including regions with underrepresented health systems, could foster broader participation, enhance inclusivity, and encourage diverse perspectives on research and care strategies.

### Strengthening policy engagement

Future symposia on multimorbidity would benefit from stronger involvement of health policy stakeholders. Encouraging participation from decision-makers and aligning symposium content with current policy debates could increase the impact of such events and promote implementation of evidence-informed strategies into healthcare systems.

Overall, the insights gained from the pre-symposium survey were invaluable for shaping the agenda of the symposium. By actively engaging stakeholders in the planning process and prioritizing topics that resonated with potential attendees, the organizing committee could create a relevant and meaningful event. By fostering a culture of continuous improvement and participant-centric planning, future editions of the International Symposium on MM have the potential to set new benchmarks in academic event management and participant engagement.

## Data Availability

The datasets used for this manuscript are available from the corresponding author on reasonable request.*
